# Disinhibition in Risky Sexual Behavior in Men, but Not Women, during Four Years of Antiretroviral Therapy in Rural, Southwestern Uganda

**DOI:** 10.1371/journal.pone.0069634

**Published:** 2013-07-19

**Authors:** Annet Kembabazi, Francis Bajunirwe, Peter W. Hunt, Jeffrey N. Martin, Conrad Muzoora, Jessica E. Haberer, David R. Bangsberg, Mark J. Siedner

**Affiliations:** 1 Department of Medicine, Mbarara University of Science and Technology, Mbarara, Uganda; 2 Department of Community Health, Mbarara University of Science and Technology, Mbarara, Uganda; 3 Department of Medicine, University of California San Francisco, San Francisco, California, United States of America; 4 Department of Epidemiology and Biostatistics, University of California San Francisco, San Francisco, California, United States of America; 5 Department of Medicine, Massachusetts General Hospital and Harvard Medical School, Boston, Massachusetts, United States of America; 6 Ragon Institute of Massachusetts General Hospital, MIT and Harvard, and Harvard Medical School, Cambridge, Massachusetts, United States of America; University of Toronto, Canada

## Abstract

**Background:**

In resource-rich areas, risky sexual behavior (RSB) largely diminishes after initiation of anti-retroviral therapy, with notable exceptions among some populations who perceive a protected benefit from anti-retroviral therapy (ART). Yet, there is limited data about long-term trends in risky sexual behavior among HIV-infected people in sub-Saharan Africa after initiation of anti-retroviral therapy.

**Methods:**

We administered questionnaires every three months to collect sexual behavior data among patients taking ART in southwestern Uganda over four years of follow-up time. We defined RSB as having unprotected sex with an HIV-negative or unknown status partner, or unprotected sex with a casual partner. We fit logistic regression models to estimate changes in RSB by time on ART, with and without adjustment for calendar year and CD4 count.

**Results:**

506 participants were enrolled between 2005 and 2011 and contributed a median of 13 visits and 3.5 years of observation time. The majority were female (70%) and median age was 34 years (interquartile range 29–39). There was a decrease in the proportion of men reporting RSB from the pre-ART visit to the first post-ART visit (16.2 to 4.3%, p<0.01) but not women (14.1 to 13.3%, p = 0.80). With each year of ART, women reported decreasing RSB (OR 0.85 per year, 95%CI 0.74–0.98, p = 0.03). In contrast, men had increasing odds of reporting RSB with each year of ART to near pre-treatment rates (OR 1.41, 95%CI 1.14–1.74, p = 0.001), which was partially confounded by changes in calendar time and CD4 count (AOR = 1.24, 95%CI 0.92–1.67, p = 0.16).

**Conclusions:**

Men in southwestern Uganda reported increasing RSB over four years on ART, to levels approaching pre-treatment rates. Strategies to promote long-term safe sex practices targeted to HIV-infected men on ART might have a significant impact on preventing HIV transmission in this setting.

## Background

Approximately 2.5 million people continue to be infected with HIV each year in sub-Saharan Africa, and the majority of new infections in the region are caused by sexual transmission between sero-discordant, heterosexual partners [1]. A thorough understanding of sexual behavior among people living with HIV/AIDS (PLWHA) will be essential to further decrease HIV incidence. In the United States and Europe, early studies of sexual behavior among PLWHA noted a “disinhibition” phenomenon after initiation of anti-retroviral therapy (ART), with increased rates of high-risk behavior in some sub-populations [2], [3], [4], [5], [6]. Yet, meta-analyses including population level data have since countered that notion [7], suggesting that increasing rates of risky behavior are largely limited to those who consider ART to be protective against transmission [8].

Recent reviews summarizing data in Sub-Saharan Africa reported similar reductions in risky sexual behavior (RSB) after ART initiation [9], [10]. However, because of the relatively short period of ART availability in the region, most of these studies are cross sectional or limited to short follow-up times, before a durable impact of ART on sexual behaviour might be realized. One recent study in Uganda with two years of post-ART follow-up documented increasing RSB to pre-ART levels [11]. To clarify associations between ART use and sexual behavior, a thorough evaluation of sexual behavior from varying regions and sufficient duration of ART exposure will be essential. We enrolled over 500 participants in a long-term cohort of PLWHA in rural Uganda from the time of ART initiation and describe here trends in sexual behavior up to four-year after ART initiation.

## Methods

### Study Procedures

We enrolled participants in the Uganda AIDS Rural Treatment Outcomes (UARTO) study from the adult HIV clinic at Mbarara regional Referral Hospital beginning in 2005. Adult PLWHA living within 20 kilometers of the clinic (to facilitate study procedures) and initiating ART were eligible. We administered surveys at baseline (pre-ART visit) and quarterly after ART initiation to collect data on sociodemographic and sexual behavior characteristics, and collected blood samples for CD4 count testing. Trained research assistants fluent in the local language performed interviews in private study rooms. They asked about number of partners, age at first sexual encounter, number of sexual partners in the past three months (primary and casual), condom use, and HIV serostatus of partners. All participants gave written informed consent to participate.

### Ethics Statement

The study procedures were approved by ethical review committees at Partners Healthcare, University of California, San Francisco, the Mbarara University of Science and Technology and the Uganda National Council for Science and Technology.

### Statistical Analyses

Our outcome of interest was risky sexual behavior (RSB). We defined RSB as having unprotected sex with a sero-discordant or unknown status partner, or unprotected sex with a casual partner in the prior three months. We fit logistic regression models to estimate changes in RSB by year of ART use, with and without adjustment for calendar year (per year) and CD4 count (per 100 cells/mm^∧^3), divided into a two-piece segmented linear model (<300 cells/mm^∧^3 and ≥300 cells/mm^∧^3) to allow for the changing slope of RSB that were apparent in graphical models. We implemented the Huber-White estimator to account for within cluster dependence from repeated measures in participants over time [12], [13], [14], [15]. We divided the analysis by gender. We censored data after 17 quarterly visits (approximately 4 years of follow-up), after which point our dataset was limited to less than 200 participants. We evaluated for a survivorship bias by refitting our logistic regression models with participants who completed at least 17 visits of observation time.

## Results

### Baseline Characteristics

A total of 506 participants (70% female) were enrolled from the time of ART initiation from November 2005– June 2011 (Table 1). Median age was 34 years (interquartile range [IQR] 29–39) and median CD4 count at the baseline visit was 132 cell/mm^∧^3 (IQR 75-202). The median age at first sexual encounter was 17 years (IQR 15–19) in women and 18 years (IQR 16–20) in men. Women reported a median of 3 lifetime sexual partners (IQR 2–4) versus men who reported a median of 6 (4–15, p<0.001)). At baseline, 49% of the entire cohort reported sexual contact in the past three months, and 16% of men and 14% of women met criteria for RSB in the past three months.

**Table 1:** Baseline Sociodemographic and Sexual Behavior Characteristics for Participants in a Longitudinal Cohort Study of PLWHA using ART.

	Male Participants	Female Participants	Total Cohort
	(n = 155)	(n = 351)	(n = 506)
**Baseline Demographic Characteristics**			
Age (median, IQR)	38 (32–43)	33 (27–38)	34 (29–39)
Married (%)	52	25	32
Baseline CD4 count (median, IQR)	116 (54–195)	139 (83–207)	132 (75–202)
Years of Observation Time (median, IQR)	3.4 (1.7–3.7)	3.5 (2.1–3.7)	3.5 (2.1–3.7)
Study Visits (median, IQR)	13 (15–17)	13 (8–15)	13 (8–15)
Enrolment Year (%)			
2005	14	12	12
2006	24	25	25
2007	34	34	34
2008	18	20	19
2009–2010	10	9	10
**Baseline Sexual Behaviour Characteristics**			
Age of First Sexual Encounter (median, IQR)	19 (17–21)	17 (15–19)	18 (16–20)
Lifetime Number of Partners (median, IQR)	6 (4–15)	3 (2–4)	3 (2–6)
Any Unprotected Sex with Unknown Status or HIV- PartnerPast 3 mos (%)	15	13	14
Any Unprotected Sex with Non-Primary Partner Past 3 months	9	7	8
**Any unprotected sex with a non-primary partner or a** **HIV-negative or Unknown Status Partner (%)**	**16**	**14**	**15**

### Longitudinal Trends in High-Risk Sexual Behavior after Antiretroviral Therapy Initiation

**Figure 1:**
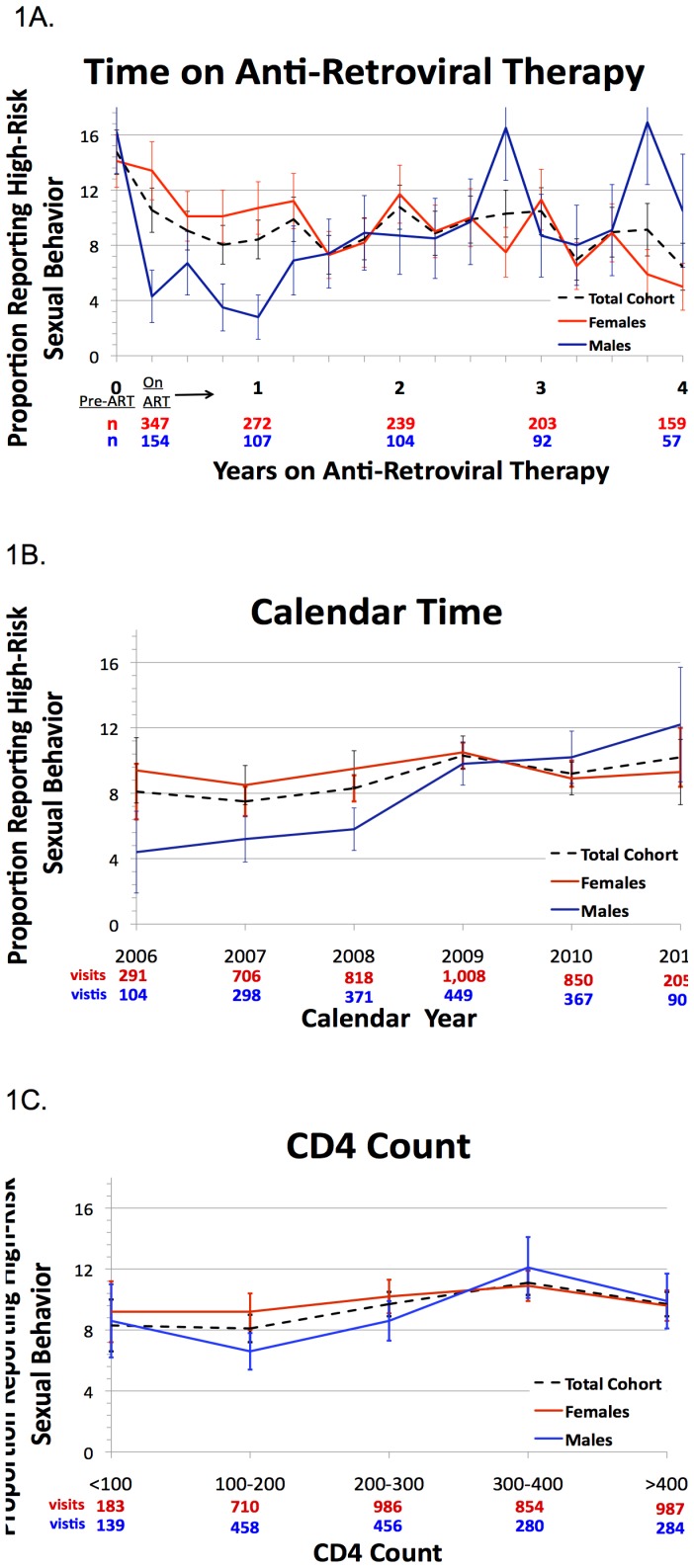
Changes in high-risk sexual behavior by time from start of ART (1A), calendar time (1B), and CD4 count (1C) among a cohort of people living with HIV/AIDS in southwestern Uganda. The red lines denote females, blue lines denote males, and black dotted lines denote the total cohort.

Men had a significant decrease in reporting RSB from the last pre-ART to the first on-ART visit (16.2% to 4.3%, p = 0.002). However, men reported increasing RSB for each year of ART use (OR = 1.41, 95%CI 1.15–1.74, p = 0.001, Figure 1A, Table 2), with each increasing calendar year (OR 1.28, 95%CI 1.06–1.55, p = 0.01, Figure 1C), and with each 100-cell increase in CD4 count <300 cells/mm^∧^3 (OR 1.85, 95%CI 1.19–2.87, p = 0.01, Figure 1B), but not ≥300 cells/mm^∧^3 (OR 0.93, 95%CI 0.72–1.19, p = 0.55). In the multivariable model adjusted for calendar year and CD4 count, the association between years on ART and RSB remained positive, but not statistically significant (AOR = 1.24 for each year of ART use, 95%CI 0.92–1.67, p = 0.11, Table 2). We found no evidence of a survivor effect in the association between RSB and years of ART use when we restricted the analysis to men who had at least four years of sexual behavior data (n = 44, 627 visits, OR 1.50, 95%CI 1.06–2.13).

**Table 2:** Logistic regression models for trends in high-risk sexual behaviour with increasing time on ART, calendar year, and CD4 count among a cohort of HIV-infected Ugandans during treatment with anti-retroviral therapy.

	Univariable Models			Multivariable Model		
	OR	95%CI	p-value	OR	95%CI	p-value
*Female Participants (3,462 quarterly study visits)*						
Years on ART	0.85	0.74–0.98	0.03*	0.72	0.55–0.93	0.01*
Calendar Year (2005–2011)	1.02	0.89–1.16	0.81	1.19	0.97–1.47	0.10
CD4 Count (per 100 cells)						
<300 cells/mm^∧^3	1.38	1.04–1.83	0.03*	1.45	1.09–1.91	0.01*
≥300 cells/mm^∧^3	0.90	0.76–1.06	0.21	0.92	0.78–1.09	0.33
*Male Participants (1,516 quarterly visits)*						
Years on ART	1.41	1.15–1.74	<0.001*	1.24	0.92–1.67	0.16
Calendar Year (2005–2011)	1.28	1.06–1.55	0.01*	1.09	0.83–1.42	0.54
CD4 Count (per 100 cells)						
<300 cells/mm^∧^3	1.85	1.19–2.87	0.01*	1.72	1.10–2.67	0.02*
≥300 cells/mm^∧^3	0.93	0.72–1.19	0.55	0.92	0.70–1.20	0.53

In contrast to men, we found no significant difference in RSB from the last pre-ART to the first on ART visit in women (14.1% to 13.4% p = 0.76). In a univariable regression model of change in RSB by year of ART, women reported decreasing RSB for each additional year of ART use (OR 0.85, 95%CI 0.74–0.98, p = 0.03, Figure 1A, Table 2). In women, we found no association between RSB and either calendar year (OR 1.02, 95%CI 0.89–1.16, p = 0.81, Figure 1B), but a significant association for those with CD4 counts <300 cells/mm^∧^3 only (OR 1.38 for each 100 cells, 1.04–1.83, p = 0.03, Figure 1C). The association between years on ART and RSB remained significant after adjusting for calendar year and CD4 count (OR 0.72, 95%CI 0.55–0.93, p = 0.01, Table 2). When we restricted the analysis to women for whom we had a full four years of data (n = 118, 1,601 study visits), we found similar, though muted associations for changes in RSB with each increasing year of ART use (OR = 0.94, 95%CI 0.81–1.10).

## Discussion

In this prospective cohort of HIV-infected individuals observed for approximately four years after ART initiation in southwestern Uganda, men reported a significant decline from the pre-ART to first on-ART visit, but then increasing RSB on ART, to rates similar to that of the pre-treatment period. Both calendar time and increasing CD4 partially confounded the association between time on ART and RSB, suggesting that improving health status on ART might be partially responsible for the association. In contrast, women reported decreasing rates of RSB with each year of ART use, which was independent of CD4 and temporal changes. Our findings add data to an emerging theory of sexual behavior trends among PLWHA on ART: namely, that while RSB decreases after initiating ART in most populations [8], [9], that some important sub-populations also appear to report increasing RSB over time, to levels at or near pre-initiation rates [10], [11], [16]. This finding is of critical importance to public health experts working to decrease HIV transmission, and raises important questions about the causes of differing sexual behavior patterns, the role of targeted interventions to sub-populations, and the relationships between RSB and drug adherence.

Increasing risk behavior with ART availability was noted early in the United States among men who have sex with men (MSM) and attributed to a “disinhibition” phenomenon [2], [3], [4], [5]. However, larger population-based studies and meta-analyses including varying risk-groups in resource rich areas noted an overall decrease in RSB over time on ART [7], [8], [17], [18], with some studies noting increasing RSB limited to high-risk groups or those who believed that ART decreased risk of transmission [16], [19], [20]. Cross-sectional and relatively short longitudinal studies in sub-Saharan Africa reported decreasing RSB in the first 6–12 months after ART initiation [21], [22], [23], [24], [25], [26], [27]. Yet, as longer follow-up time became available, studies began reporting increasing RSB with longer ART exposures. A study in Uganda followed participants for two years after ART initiation and reported a decline in RSB among both sexes from the pre-ART to the post-ART period [28], but also an absolute increase in unprotected sex with HIV-negative or unknown status partners from 6 months to 24 months-post ART in both men and women (9% to 13% and 8% to 15% respectively). A separate seven year cohort in Uganda including participants in both the pre and post-ART periods also found significant decreases in RSB in the early post-ART period followed by a regression to pre-ART baseline levels in both men and women after two to three years of ART use [11]. We report here similar findings of a “rebound” phenomenon of increasing RSB among men, but not women, during four years of ART use in southwestern Uganda.

Further discernment of long-term trends and causes of increasing RSB will serve multiple purposes. Measurement of the association between RSB and ART adherence is a priority. Though HIV transmission can be reduced among discordant heterosexual couples when one of the partners is using ART [29], [30], its impact is less clear for other routes of transmission [31] and is reduced by non-adherence. Although we did not ask participants about reasons for their sexual behavior practices, formative work to determine the causes of increasing risky sexual behavior during ART use should be pursued to help target interventions that mitigate risky behavior practices. As noted previously, some studies in developed settings have suggested that knowledge or “optimism” about the effects of ART contributes to “disinhibition” in sexual behavior [19], [20]. A prospective study from Mozambique reported similar findings, namely that risky sexually behavior was strongly correlated with perceptions that ART was protective against transmission [32]. These findings would support the practice of repeated sexual behavior counselling during longterm ART provision, focusing on risks of other sexually transmitted diseases and family planning considerations. Though multiple interventions for reducing RSB have been developed and tested with some success [33], they have predominantly focused on men who have sex with men populations in resource-rich areas [34], with some additional examples from sub-Saharan Africa that have been largely confined to studies of clinic-based interventions [35].

Our study was limited to four years of follow-up. As ART availability continues to expand in sub-Saharan Africa, and because our estimates suggest fluid trends in reporting of RSB up to four years, continued observance of the long-term trends in RSB is recommended. Moreover, we primarily focused on describing global trends of RSB and did not evaluate time varying correlates of RSB aside from CD4 count and calendar time. Future studies should measure predictors of RSB to identify at-risk sub-populations and whether adherence changes correlate with RSB. Finally, our data are limited to self-report of RSB. The validity of self-reporting sensitive information can be poor due to motivational biases, cognitive factors, and privacy concerns, with a predicted bias towards under-reporting [36]. Such a bias in our study would have lead to underestimating associations between years of ART use and RSB.

In summary, while women in rural Uganda report decreasing RSB over four years after initiation of ART, men reported an initial decrease followed by increased RSB during time on ART to pre-baseline levels. Future research should focus on associations between RSB and ART adherence as well as correlates of the heterogeneous patterns of risk. Intervention strategies that are aimed specifically at high-risk groups will be needed to effectively impact RSB and HIV transmission in the region.
